# Community-supported teaching on the topic of transgender identity in undergraduate medical education – a pilot project

**DOI:** 10.3205/zma001640

**Published:** 2023-09-15

**Authors:** Matthias Besse, Jörg Signerski-Krieger, Hannah Engelmann, Né Fink, Isabel Methfessel, Michael Belz

**Affiliations:** 1 University Medical Center Göttingen, Clinic for Psychiatry and Psychotherapy, Göttingen, Germany; 2 Trans*Counseling Göttingen c/o Queer Center Göttingen, Göttingen, Germany

**Keywords:** community-supported teaching, transgender identity, undergraduate medical education, teaching evaluation, gender incongruence

## Abstract

**Introduction and objectives:**

Future physicians are insufficiently prepared for the topic of transgender identity during their studies. Relevant courses during undergraduate medical education are heterogeneous and not widely established within the curricula. At our university, we investigated if students' knowledge of transgender identity could be increased through medical specialist teaching and teaching delivered by representatives of the trans* community (community-supported teaching).

**Methods:**

During summer semester 2021 (SS21), the knowledge level on transgender identity of 134 medical students in their fifth clinical semester was evaluated (phase 1). In addition, knowledge gain on gender incongruence through the module “psychiatry” was retrospectively surveyed across two dimensions: 1. diagnostic criteria, 2. treatment/care.

During winter semester 2021/22 (WS 21/22), a 90-minute seminar on transgender identity was held either community-supported or by medical specialists (phase 2). Following the psychiatry exam, a re-evaluation was carried out by 115 students (phase 3).

**Results:**

The students in SS21 did not feel sufficiently educated in the topic of transgender identity through their studies, but rated the relevance of the topic for their later profession as high. Learning gain improved after the introduction of the seminar in WS21/22 compared to the previous semester (both dimensions *p*<.001). Community-supported and specialist teaching achieved equivalent results.

**Conclusion:**

One 90-minutes seminar led to a significant learning gain regarding the topic of transgender identity. Community-supported teaching is a promising way to impart knowledge in a qualified manner: Medical faculties should use this form of teaching to convey established knowledge to students in future curricula.

## Zusammenfassung

**Zielsetzungen:**

Zukünftige Ärzt*innen werden im Studium unzureichend auf das Thema Transidentität vorbereitet. Lehrveranstaltungen sind im internationalen Vergleich heterogen und nicht an allen Universitäten etabliert. An unserer Universität wurde untersucht, ob sich das Wissen der Studierenden zu diesem Thema durch fachärztliche Lehre sowie durch Lehre, die von Vertreter*innen der Trans*Community gehalten wurde (communitygestützte Lehre) in ähnlicher Form steigern lässt.

**Methoden:**

Im Sommersemester 2021 (SS21) wurde der Wissensstand zu Transidentität von 134 Medizinstudierenden im fünften klinischen Semester evaluiert (Phase 1). Zudem wurde der Lernzuwachs zu Genderinkongruenz durch das Modul Psychiatrie retrospektiv in zwei Dimensionen erfragt: 1. Diagnosekriterien, 2. Behandlung/Begleitung.

Im Wintersemester 2021/22 (WS 21/22) wurde ein 90-minütiges Seminar zum Thema Transidentität fachärztlich oder communitygestützt durchgeführt (Phase 2). Im Anschluss an die Klausur Psychiatrie erfolgte eine erneute Evaluation durch 115 Studierende (Phase 3).

**Ergebnisse:**

Die Studierenden im SS21 fühlten sich durch ihr Studium nicht ausreichend im Thema Transidentität ausgebildet, schätzten die Relevanz des Themas für ihren späteren Beruf aber hoch ein. Der Lernzuwachs verbesserte sich nach Einführung des Seminars im WS 21/22 im Vergleich zum vorherigen Semester (beide Dimensionen *p*<,001). Die communitygestützte und die fachärztliche Lehre erzielte gleichwertige Lernzuwächse.

**Schlussfolgerung:**

Ein 90-minütiges Seminar führte bei Studierenden zu einem signifikanten Lernzuwachs bezüglich Transidentität. Communitygestützte Lehre ist hierbei eine gute Möglichkeit, Wissen zu vermitteln. Medizinische Fakultäten sollten diese Form der Lehre nutzen, um in zukünftigen Curricula etabliertes Wissen gezielt an Studierenden zu vermitteln.

## Introduction

In Germany, according to the Basic Constitutional Law, no person may experience disadvantages because of the gender [https://www.gesetze-im-internet.de/gg/]. Recently, several steps have been taken towards equality for transgender people: For example, in 2017, the Federal Ministry of Family Affairs advocated for a new regulation of the Transsexuals Act [1]. In the General Act on Equal Treatment, protection against discrimination of transgender people is explicitly mentioned as a goal [2].

Medically, there has also been a rethinking on the topic of transgender identity. After five forms of “gender identity disorders” were distinguished in the tenth version of the International Classification of Diseases (ICD-10) ([3], 10^th^ revision), the chapter was deleted from the area of personality disorders in ICD-11 and replaced by the chapter “conditions related to sexual health” and renamed from “gender identity disorders” to “gender incongruence” ([3], 11^th^ revision). This move was associated by representatives of the trans*community with the hope that this would lead to destigmatization and improvement in care [4]. Despite these developments, transgender people are often faced with discrimination and exclusion [5]. The proportion of mental illnesses and suicides is increased compared to the rest of the population [6].

Teaching in medical school on trans-specific topics is often limited and the knowledge of future physicians is characterized by uncertainties [7], [8], [9], [10]. In recent years, there have been efforts in several countries to standardize teaching of transgender identity in medical school. Among other things, competencies concerning sexual health that should be acquired within undergraduate medical education have been formulated [11] or a curriculum for the medical care of transgender people has been developed [12], since teaching so far differs considerably between universities [13], [14]. The literature describes the successful implementation of different teaching formats in medical studies. These range from a single lecture [15] to multi-hour [16] or multi-day curricula [17] to increase medical students’ knowledge of health problems in people with gender incongruence. Several studies report additional benefits when members of the “Lesbian, Gay, Bisexual, Transgender and Queer” (LGBTQ) community are included in courses [18], [19] or when students are introduced to the topic using simulation patients [20]. In *peer-assisted teaching*, students are taught by individuals who are not themselves professional teachers of the subject (e.g., affected individuals or fellow students). In medical studies, this is mainly used in tutorials [21] or in teaching practical skills [22], [23]. Learning gain and satisfaction are on a similar level compared to classical teaching [21], [22], [23], [24].

Our study will contribute to answer the following research questions:

1. What is the knowledge level of students on transgender identity?
2. Can students’ knowledge be significantly increased by implementing topic-specific teaching in the form of a 90-minute seminar?
3. Does peer-assisted teaching (in this case: representatives of the trans* community, hence referred to as *“community-supported teaching”* in the following) lead to equivalent learning gain if compared to specialist teaching?

Prior to the implementation of the course, knowledge level on transgender identity and knowledge gain among students of the summer semester 2021 (SS21) were surveyed (phase 1), before the implementation of a 90-minute seminar on transgender identity in the winter semester 2021/2022 (WS21/22; phase 2: community-supported or specialist teaching). Finally, an evaluation was conducted in WS21/22 (phase 3).

## Material and methods

### Phase 1: Knowledge level survey in summer semester 2021

As part of the module evaluation, the survey of the students of the fifth clinical semester was conducted in SS21 following the psychiatry exam. A questionnaire, created and evaluated with EvaSys (Electric Paper Evaluation Systems) was distributed to all 155 students. For the evaluation, five items were added to the existing evaluation questionnaire (see table 1 (Tab. 1)), which were formulated as statements (e.g., “I can name the criteria for gender identity disorder”). Since the ICD-11 was not yet officially used at the time of questionnaire development, the terminology “gender identity disorder” from the ICD-10 was used. All statements could be answered using a six-point numerical scale with two anchors (1=“strongly agree” to 6=“strongly disagree”). Three items nos. 1-3 were used to assess* knowledge level* on the topic of transgender identity. For two paired items (nos. 4-5), students assessed their subjective *learning gain* by providing their retrospective self-assessment at the beginning of the module (pre-teaching), and their self-assessment at the time of the survey (post-teaching).

In order to maintain anonymity, no data were collected or analyzed in the course of the module evaluation that would allow conclusions to be drawn about individuals. Teaching on the topic of transgender identity did not take place in SS21.

### Phase 2: Implementation of the seminar in winter semester 2021/2022

The students from WS21/22 represented a completely new cohort – no students from SS21 participated in phases 2 and 3. In WS21/22, one seminar of 90-minute duration on the topic of transgender identity was held in six small groups (A-F) of no more than 25 students. Due to the Corona pandemic, courses with mandatory attendance in WS21/22 were delivered digitally using the “BigBlueButton” web conferencing system.

Groups A and D were taught by a specialist in psychiatry with additional qualification in sexual medicine, groups B and E by a specialist in psychiatry (*specialist teaching*). For groups C and F, two lecturers from the local “Queer Center” were recruited (*community-supported teaching*). Both lecturers, who themselves live transident, have been involved in counseling of trans* persons for many years, regularly give lectures on the topic of transgender identity and publish scientific articles in this field.

All lecturers were informed about the learning objectives and the structure of the seminar. An interactive discussion had to be part of the seminar, with further design left to the lecturers.

### Phase 3: Learning gain evaluation in winter semester 2021/2022

The evaluation of the new course was carried out in the same way as in phase 1. As part of the module evaluation following the psychiatry exam of WS21/22, the questionnaire was again distributed and supplemented by the questions described in table 1 (Tab. 1) (distribution to all 138 students). In addition, students were asked to indicate their seminar group. As in SS21, no personal data were collected or evaluated.

### Statistical analysis

Data analysis was performed using IBM SPSS^®^ software, version 29. Means (*M*), mean differences (*M_Diff_*), standard deviations (*SD*), and Pearson-correlations (*r*) were created for descriptive presentation. Delta values for estimated learning gain were calculated (Δ=(post-teaching)-(pre-teaching)); negative values indicated learning gain.

In order to analyze differences in assessed *knowledge level* about transgender identity between the two semesters SS21 and WS21/22 beyond descriptive presentation, t-tests including effect sizes (*d_emp_*) were calculated for independent samples for items nos. 1-3 (see table 1 (Tab. 1)). To analyze *learning gain* in the field of transgender identity, two general linear models (GLM) for repeated measures were created for the pairwise items nos. 4-5 (see table 1 (Tab. 1)), with inclusion of pre- and post-teaching self-assessment as a two-level within-subject factor (“retrospective” vs. “current self-assessment”). Semester was included as a two-level between-subjects factor in the respective GLM (SS21 vs. WS21/22). To identify differences in learning gain between the two semesters, the interaction effect of the two factors was tested for significance for both GLMs (semester × learning gain). Furthermore, differences in learning gain between *community-supported teaching* and *specialist teaching* were analyzed exclusively for WS21/22 using two additional GLMs; they included self-assessment of learning gain as a two-level within-subject factor, and teaching form (community-supported vs. specialist) as a two-level between-subject factor, including testing for interaction of both factors.

Because of α-error inflation (starting from α=0.05, two-sided testing), *p*-values were adjusted by Bonferroni method for a total of 7 statistical tests (4×GLM, 3×t tests; *p_adj_*=*p_emp_*×7). Adjustment was also made for all reported pairwise comparisons within each GLM. Students could provide incomplete information in the questionnaire or omit items completely – see degrees of freedom for each model, or statistical test, and information in figures and tables for the included cases.

## Results

### Knowledge level and learning gain in summer semester 2021

**Figure 1:**
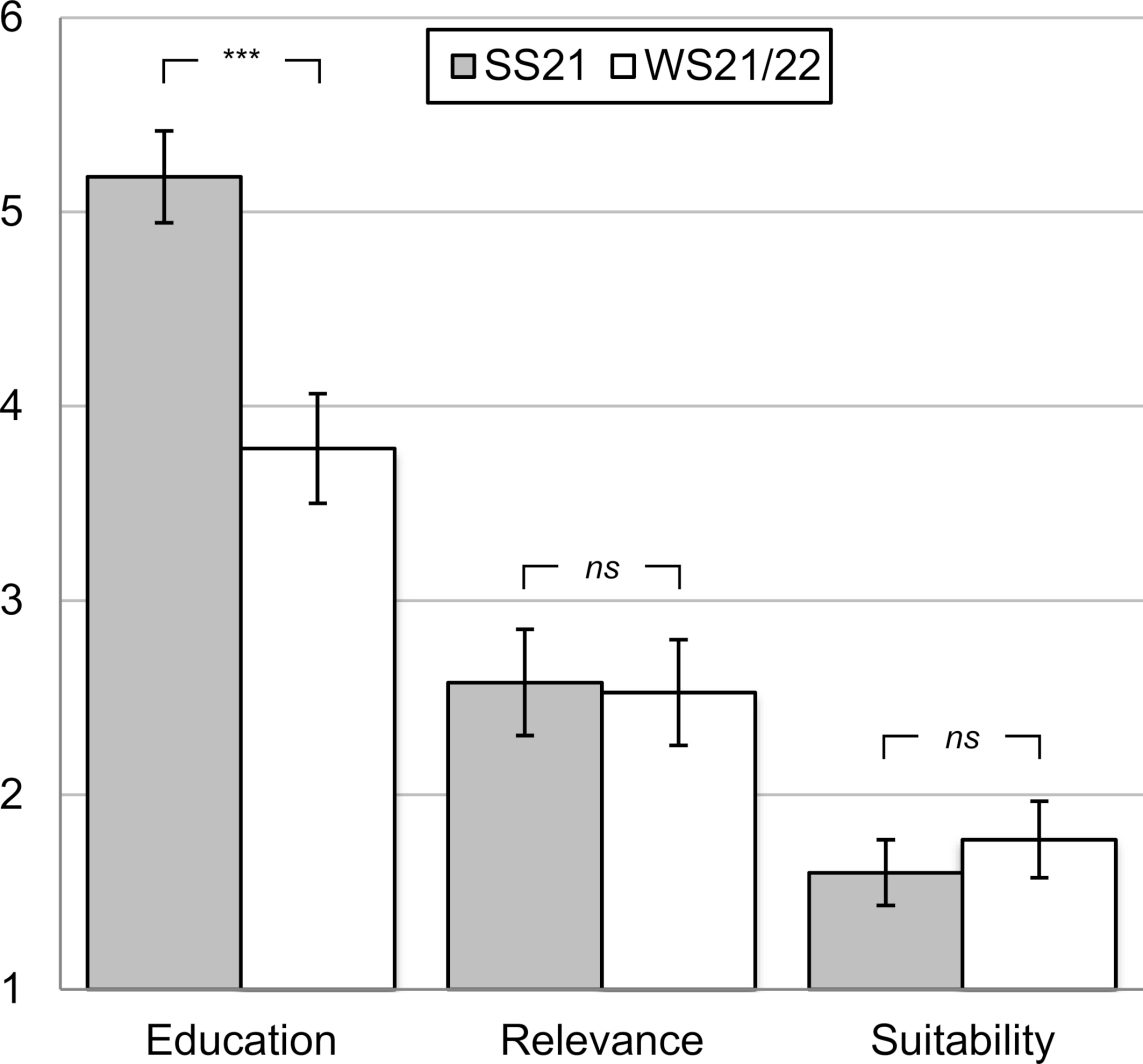
Knowledge level in the field of transgender identity. Mean values with 95% confidence intervals for students' self-assessment on a 6-point numerical scale with two anchors (1=“strongly agree” to 6=“strongly disagree”) on the following items: (1) *education* in the course of study regarding gender identity disorders, (2) *relevance* of the topic for later professional life, (3) *suitability* of the module for teaching the topic. For all item formulations, see table 1; *SS21*=Summer semester 2021 (*n*=115 to 128); *WS21/22*=Winter semester 2021/22 (*n*=106 to 109).

Of 155 students in SS21, *n*=134 (86.5%) provided information. Students indicated on the numerical scale that they did not feel adequately educated in gender identity disorders through their studies (*M*=5.18, *SD*=1.37), tended to rate the topic as relevant to their future careers (*M*=2.58, *SD*=1.53), and felt that the psychiatry module was most appropriate to address the topic (*M*=1.60, *SD*=0.93; see figure 1 (Fig. 1)).

The students indicated a moderate learning gain in the field of gender identity disorders already in SS21, although no teaching had taken place on the topic: It was Δ=-0.44 (*M_pre_*=5.07, *M_post_*=4.62) for “diagnostic criteria”, and Δ=-0.27 (*M_pre_*=5.26, *M_post_*=4.99) for “treatment and care”. Compared to other content taught in SS21 (e.g., antidepressants), the increase in the field of gender identity disorders was numerically considerably lower (for comparison, learning gain for other content taught in SS21 ranged from Δ=-1.68 to -2.67; see also [25]).

### Modified teaching in winter semester 2021/22

Of 138 students in WS21/22, n=115 (83.3%) provided information. Table 2 (Tab. 2) shows an overview of the correlations between questionnaire items and semester affiliation.

Considering *knowledge level*, there was a difference between the two semesters. While students in SS21 tended to disagree with the statement that they had received sufficient training in the field of gender identity disorders through their studies (value >5, see above), the students of WS21/22 achieved a value of *M*=3.78 (*SD*=1.48) and felt significantly better trained (*t*(232)=7.45,* p*<.001, *d_emp_*=0.99, *r*=-0.442; see figure 1 (Fig. 1) and table 2 (Tab. 2)). The relevance of the topic to everyday professional life and the tendency of the module to be suitable for teaching were not rated differently in WS21/22 compared to SS21 (*t* from 0.26 to 1.29, *ns*, *d_emp_* from 0.03 to 0.17; see figure 1 (Fig. 1) and table 2 (Tab. 2)).

A numerically larger *learning gain* was found in WS21/22 compared to SS21. This is reflected in the correlations between semester and learning gain (see table 2 (Tab. 2): *r* from -0.483 to -0.607, all *p*<.001). Learning gain in WS21/22 was Δ=-1.61 (*M_pre_*=4.55, *M_post_*=2.94) for “diagnostic criteria” and Δ=-1.74 (*M_pre_*=4.57, *M_post_*=2.83) for “treatment and care”. The general amount of learning gain in the field of transgender identity compared to regularly taught content in WS21/22 was equivalent (for comparison, learning gain for regularly taught content in WS21/22 ranged from Δ=-1.71 to -2.56).

**Figure 2:**
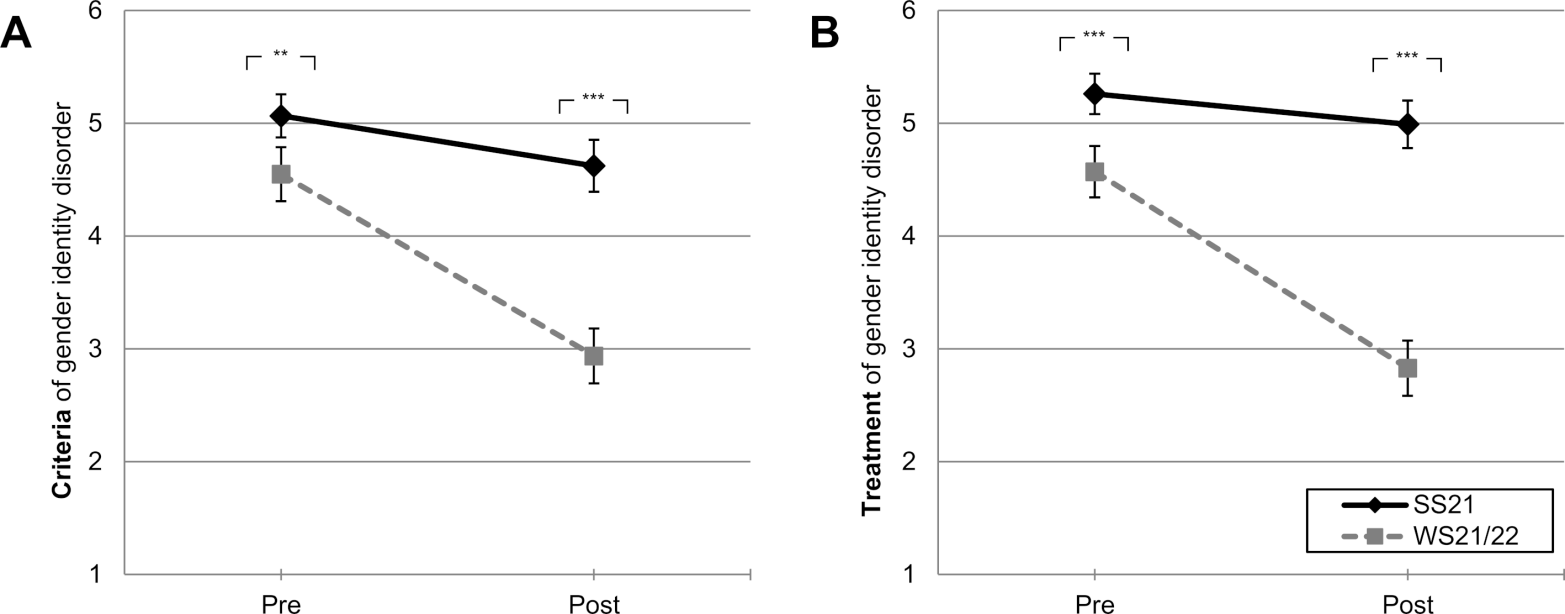
Learning gain. Mean values with 95% confidence intervals and Bonferroni-corrected pairwise comparisons of students’ self-assessed learning gain on a 6-point numerical scale with two anchors (1=“strongly agree” to 6=“strongly disagree”); “at the beginning of the module” (*pre*-teaching) vs. “currently” (*post*-teaching), for (A) diagnostic *criteria* of gender identity disorder, (B) *treatment* and care of gender identity disorder (see tab. 1 for all item formulations); differentiated by *SS21* (summer semester 2021, *n*=119 to 122) and *WS21/22* (winter semester 2021/22, *n*=112 to 113). *p<.05; **p<.01; ***p<.001, *ns*=not significant.

In the general linear model for “diagnostic criteria of gender identity disorder” (see figure 2 A (Fig. 2)), a significant repeated measures effect was found describing a general learning gain for the overall sample (GLM: *F*(1,233)=219.13, *p*<.001, partial η^2^=0.49). Furthermore, a significant between-groups effect was found between both semesters (GLM: *F*(1,233)=55.67, *p*<.001, partial η^2^=0.19): Both at the retrospectively assessed time point (pre-teaching) and at the current time point after teaching, WS21/22 students estimated their knowledge to be greater than SS21 students. A significant interaction effect was also found (GLM: *F*(1,233)=70.91, *p*<.001, partial η^2^=0.23): As shown in figure 2 A (Fig. 2), learning gain increased to a greater extent for students in WS21/22 than for students in SS21 (pre-teaching: *M_WS21/22_*=4.55, *M_SS21_*=5.04, *M_Diff_*=0.49, *p*=.009 ; post-teaching: *M_WS21/22_*=2.94, *M_SS21_*=4.63, *M_Diff_*=1.70, *p*<.001).

Comparable results were found for the domain “treatment and care of gender identity disorder” (see figure 2 B (Fig. 2)). In addition to a learning gain for the total sample (repeated measures effect; GLM: *F*(1,229)=248.41, *p*<.001, partial η^2^=0.52) and a between-groups effect between both semesters (GLM: *F*(1,229)=100.57, *p*<.001, partial η^2^=0.31), significant differences in the course of learning gain were found (interaction effect; GLM: *F*(1,229)=133.26, *p*<.001, partial η^2^=0.37). It improved more for WS21/22 students than for SS21 students (pre-teaching: *M_WS21/22_*=4.57, *M_SS21_*=5.25, *M_Diff_*=0.68, p<.001; post-teaching: *M_WS21/22_*=2.83, *M_SS21_*=4.97, *M_Diff_*=2.14, p<.001).

In summary, students in WS21/22 showed significantly greater learning gain than students in SS21 in both areas assessed after implementation of topic-specific teaching.

### Comparison of community-supported teaching to specialist teaching

There were *n*=105 students in WS21/22 who provided information on who taught the seminar in each case. Response rates approximately represent the 2/3 distribution to specialist teaching (*n*=70) and 1/3 distribution to community-supported teaching (*n*=35, 1/3).

Marginal numerical differences emerged between the two types of teaching considering knowledge level: Students felt they had received equivalent training in gender identity disorders in both forms of teaching (M*_Diff_*=0.34), estimated relevance to their future careers (M*_Diff_*=0.53), and rated the appropriateness of the module similarly (*M_Diff_*=0.23).

**Figure 3:**
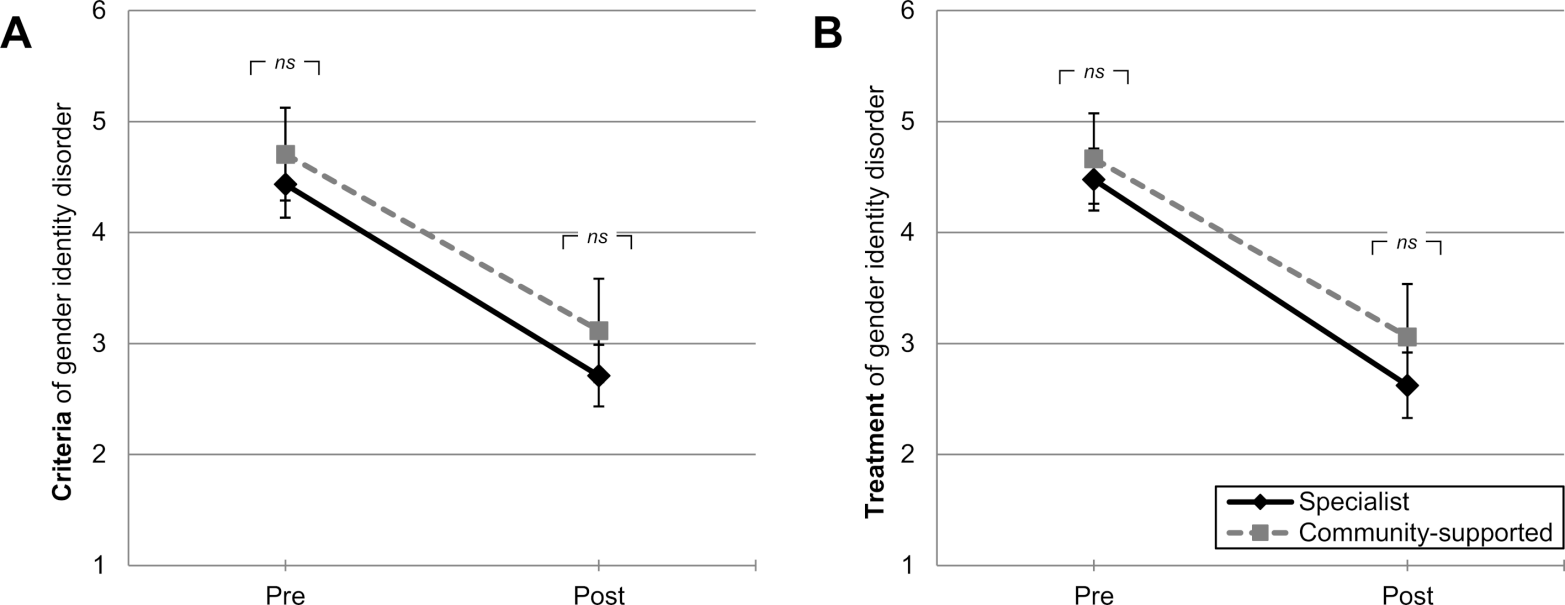
Learning gain depending on teaching form. Mean values with 95% confidence intervals and Bonferroni-corrected pairwise comparisons of learning gain, self-assessed by students in the winter semester 2021/22 on a 6-point numerical scale with two anchors (1=“strongly agree” to 6=“strongly disagree”); “at the beginning of the module” (*pre*-teaching) vs. “currently” (*post*-teaching), for (A) diagnostic *criteria* of gender identity disorder, (B) *treatment and care* of gender identity disorder (see tab. 1 for all item formulations); differentiated by *specialist* teaching (n=69) and *community-supported* teaching (*n*=33 to 34). *p<.05; **p<.01; ***p<.001, *ns*=not significant.

For *learning gain* in “diagnostic criteria of gender identity disorder” (see figure 3 A (Fig. 3)), a general repeated measures effect was found describing overall learning gain within WS21/22 regardless of teaching form (GLM: *F*(1,101)=180.34, *p*<.001, partial η^2^=0.64). Furthermore, neither a significant between-groups effect between teaching forms (GLM: *F*(1,101)=2.12, *ns*), nor a significant interaction effect was found (GLM: *F*(1,101)=0.31, *ns*, all pairwise comparisons ns). For “treatment and care” (see figure 3 B (Fig. 3)) a significant learning gain was found for the entire WS21/22 (repeated measures effect; GLM: *F*(1,100)=177.77, *p*<.001, partial η^2^=0.64), again independent of teaching form (between-groups effect; GLM: *F*(1,100)=1.87, *ns*) and with a similar pattern (interaction effect; GLM:* F*(1,100)=0.92, *ns*, all pairwise comparisons *ns*).

In summary, learning gain between community-supported teaching and specialist teaching can be considered statistically equivalent.

## Discussion

This study examined medical students’ knowledge level of transgender identity in the fifth clinical semester (SS21). Subsequently, a mandatory 90-minute seminar was implemented for WS21/22 in a new cohort of students. In conclusion, we assessed whether the adaptation resulted in improved learning gain.

### Knowledge level in the field of transgender identity

The students of SS21 did not feel sufficiently educated in the topic of transgender identity, in line with existing publications [7], [9], [10]. In addition, the highly rated professional relevance of the topic found here was likewise reported in a Canadian study conducted at nine universities. There, >95% of the students assessed the topic of transgender identity as important for their later professional life [13].

The knowledge level is also reflected in the assessed learning gain: The students of SS21 did not see themselves in a position to name diagnostic criteria of gender identity disorder, or to treat or care for those patients. The assessment of the students of SS21 changed only slightly after completion of the psychiatry module, since there was no specific teaching on the topic in this semester. The present study cannot answer why there was nevertheless a moderate learning gain in SS21, which was, however, considerably lower if compared to other learning objectives of the psychiatry module. It is possible that the topic of transgender identity was addressed by individual students in the courses of SS21 in the context of the thematization of the F-axis of the ICD-10, or that students worked out the topic themselves in self-study through increased awareness.

### Learning gain after adaptation of teaching

Participation in the implemented 90-minute seminar in WS21/22 potentially led to a significant teaching improvement on transgender identity: WS21/22 students felt significantly better educated than SS21 students in terms of knowledge about diagnostic criteria and about treatment/care of patients with transgender identity. Self-assessed learning gain in WS21/22 was comparable to other learning objectives of the psychiatry module [25].

It is worth mentioning in this context that this effect could be achieved by a 90-minute seminar. Our study supports the finding that even single courses can significantly increase knowledge about transgender identity [24], [25], [26], [27], [28]. This gives medical schools the opportunity to supplement their teaching catalog at a manageable cost. In which section of the medical curriculum such a course would have to be integrated has to be the subject of future discussions. In our study, students indicated that they thought the psychiatry module was most appropriate in this matter. However, studies on improving the teaching of transgender identity can be found for other departments as well [20], [28], [29], [30]. Given the numerous specialties involved in treatment, an interdisciplinary course would address the complexity of the topic.

### Learning gain through community-supported teaching

Our study provides preliminary evidence that community-supported teaching can lead to equivalent learning gain compared with specialist teaching: Students felt equivalently prepared about transgender identity. This finding is consistent with the results of other studies, most of which examined the teaching of practical skills. A student-led tutorial teaching basic clinical skills resulted in a high level of satisfaction among both teachers and learners [31]. Similar results were obtained in a study comparing a student-led emergency medicine seminar with physician-led groups [24]. Examinations such as ultrasound could also be taught in this manner [22].

Involving members of the LGBTQ community in teaching has been described in the literature [18], [19]. However, taking the role of sole lecturer is a unique feature of our study. According to statistics from the German Medical Association, only 39 physicians in Germany held the additional training in sexual medicine as of December 31, 2020 [32]. The inclusion of affected persons from the community represents an opportunity to acquire additional expertise for university teaching or to expand it with a new perspective. Even though there is a lack of reliable data, a didactic background of the lecturers from the community, as in the case of the present study, seems to be an advantage. In our view, the minimum requirement should be didactic training by experienced teachers at the respective university.

### Limitations and strengths

Students’ learning gain was assessed at a single point in time following the exam – this is a post-hoc survey with retrospective self-assessment, which is more susceptible to recall bias. Moreover, additional predictors (e.g., age) and differentiation by subgroups (e.g., gender) would allow for further interpretation of the data, or a check on the validity of the estimated learning gain (e.g., through final grades instead of self-assessment). Unfortunately, it was not possible to collect this kind of personal information due to anonymity requirements.

Furthermore, data show that in WS21/22 students retrospectively assessed their knowledge level in the field of transgender identity better than students in SS21 – even before the adaptation of teaching (see figure 2 (Fig. 2)). One possible interpretation is that students in WS21/22 had a higher affinity for the topic and benefited more from the adapted teaching in terms of a limited representative semester. At the same time, a significant learning gain is harder to achieve for students who have a better baseline level of knowledge [33] – this would argue for the effectiveness of adapted teaching.

A standardized schedule for the lecturers would have increased the comparability between the groups in terms of internal validity, but at the same time would have restricted the individual freedom of design. For the present project, lecturers were specifically given such freedom in shaping the seminar, whereby learning objectives and basic structure (including time-frame, technology, interaction with students) were predetermined. In this way, personal knowledge as well as previous experience could be largely incorporated into the teaching process.

## Conclusion

Teaching on trans-specific topics in medical school is limited despite low levels of knowledge on the part of students. The use of short seminars is straightforward and can increase students’ knowledge of transgender identity, highlight misconceptions, open up questions and provide pointers to further knowledge. In the medium term, interdisciplinary teaching should be used as part of a curriculum. Community-supported teaching can play a role in bringing knowledge about transgender identity to universities.

## Competing interests

The authors declare that they have no competing interests. 

## Figures and Tables

**Table 1:**
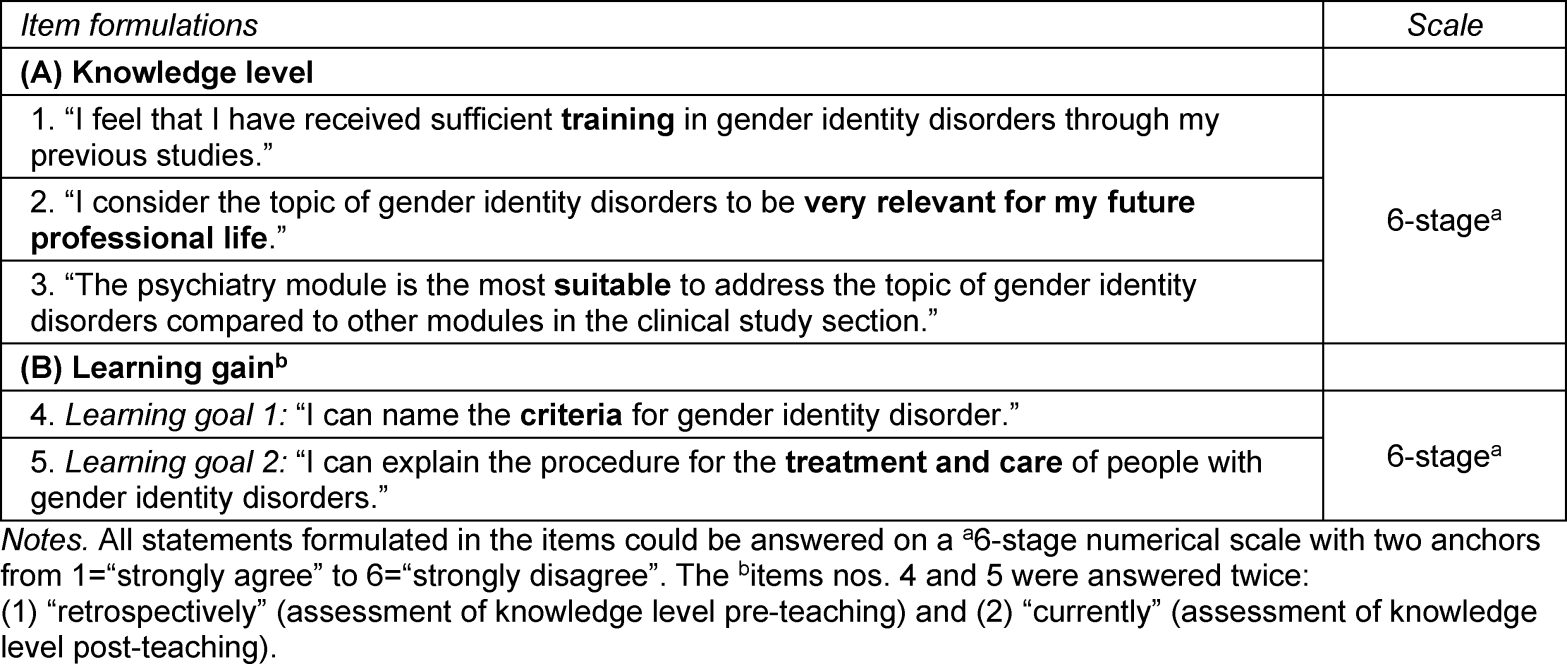
Formulation of teaching evaluation items

**Table 2:**
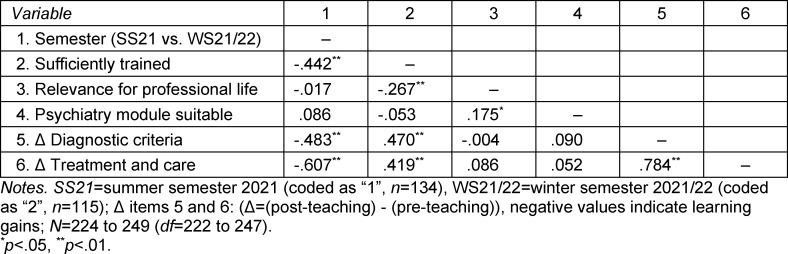
Correlations

## References

[1] Bundesministerium für Familie, Senioren, Frauen und Jugend (2017). Schutz und Akzeptanz von geschlechtlicher Vielfalt. Schlussfolgerungen des Bundesministeriums für Familie, Senioren, Frauen und Jugend aus der Arbeit der Interministeriellen Arbeitsgruppe Trans- und Intersexualität.

[2] Antidiskriminierungsstelle des Bundes (2021). AGG-Wegweiser. Erläuterungen und Beispiele zum Allgemeinen Gleichbehandlungsgesetz.

[3] Bundesinstitut für Arzneimittel und Medizinprodukte (2022). ICD-10-GM. Internationale statistische Klassifikation der Krankheiten und verwandter Gesundheitsprobleme.

[4] Bundesverband Trans* (2018). BVT* begrüßt ICD 11 der WHO: Verbesserung der Transgendergesundheitsversorgung in Aussicht.

[5] Deutscher Ethikrat (2012). Intersexualität Stellungnahme.

[6] Bundesverband Trans* (2017). Trans* Gesundheitsversorgung - Forderungen an die medizinischen Instanzen und an die Politik.

[7] Arthur S, Jamieson A, Cross H, Nambiar K, Llewellyn CD (2021). Medical students' awareness of health issues, attitudes, and confidence about caring for lesbian, gay, bisexual and transgender patients: a cross-sectional survey. BMC Med Educ.

[8] Campbell MH, Gromer J, Emmanuel MK, Harvey A (2022). Attitudes Toward Transgender People Among Future Caribbean Doctors. Arch Sex Behav.

[9] Green AR, Chun MBJ, Cervantes MC, Nudel JD, Duong JV, Krupat E, Betancourt JR (2017). Measuring Medical Students' Preparedness and Skills to Provide Cross-Cultural Care. Health Equity.

[10] Liang JJ, Gardner IH, Walker JA, Safer JD (2017). Observed Deficiencies in Medical Student Knowledge of Transgender and Intersex Health. Endocr Pract.

[11] Bayer CR, Eckstrand KL, Knudson G, Koehler J, Leibowitz S, Tsai P, Feldman JL (2017). Sexual Health Competencies for Undergraduate Medical Education in North America. J Sex Med.

[12] Ellaway RH, Thompson NL, Temple-Oberle C, Pacaud D, Frecker H, Jablonski TJ, Demers J, Mattatall F, Raiche J, Hull A, Jalil R (2022). An undergraduate medical curriculum framework for providing care to transgender and gender diverse patients: A modified Delphi study. Perspect Med Educ.

[13] Chan B, Skocylas R, Safer JD (2016). Gaps in Transgender Medicine Content Identified Among Canadian Medical School Curricula. Transgend Health.

[14] Obedin-Maliver J, Goldsmith ES, Stewart L, White W, Tran E, Brenman S, Wells M, Fetterman DM, Garcia G, Lunn MR (2011). Lesbian, gay, bisexual, and transgender-related content in undergraduate medical education. JAMA.

[15] Wahlen R, Bize R, Wang J, Merglen A, Ambresin AE (2020). Medical students' knowledge of and attitudes towards LGBT people and their health care needs: Impact of a lecture on LGBT health. PLoS One.

[16] Minturn MS, Martinez EI, Le T, Nokoff N, Fitch L, Little CE, Lee RS (2021). Early Intervention for LGBTQ Health: A 10-Hour Curriculum for Preclinical Health Professions Students. MedEdPORTAL.

[17] Thompson H, Coleman JA, Iyengar RM, Phillips S, Kent PM, Sheth N (2020). Evaluation of a gender-affirming healthcare curriculum for second-year medical students. Postgrad Med J.

[18] Levy A, Prasad S, Griffin DP, Ortega M, O'Malley CB (2021). Attitudes and Knowledge of Medical Students Towards Healthcare for Lesbian, Gay, Bisexual, and Transgender Seniors: Impact of a Case-Based Discussion With Facilitators From the Community. Cureus.

[19] Noonan EJ, Sawning S, Combs R, Weingartner LA, Martin LJ, Jones VF, Holthouser A (2018). Engaging the Transgender Community to Improve Medical Education and Prioritize Healthcare Initiatives. Teach Learn Med.

[20] Vance SR, Dentoni-Lasofsky B, Ozer E, Deutsch MB, Meyers MJ, Buckelew SM (2021). Using Standardized Patients to Augment Communication Skills and Self-Efficacy in Caring for Transgender Youth. Acad Pediatr.

[21] Karamaroudis S, Poulogiannopoulou E, Sotiropoulos MG, Kalantzis T, Johnson EO (2020). Implementing Change in Neuroanatomy Education: Organization, Evolution, and Assessment of a Near-Peer Teaching Program in an Undergraduate Medical School in Greece. Anat Sci Educ.

[22] Ben-Sasson A, Lior Y, Krispel J, Rucham M, Liel-Cohen N, Fuchs L, Kobal SL (2019). Peer-teaching cardiac ultrasound among medical students: A real option. PLoS One.

[23] Gradl-Dietsch G, Menon AK, Gursel A, Gotzenich A, Hatam N, Aljalloud A, Schrading S, Hölzl F, Knobe M (2018). Basic echocardiography for undergraduate students: a comparison of different peer-teaching approaches. Eur J Trauma Emerg Surg.

[24] House JB, Choe CH, Wourman HL, Berg KM, Fischer JP, Santen SA (2017). Efficient and Effective Use of Peer Teaching for Medical Student Simulation. West J Emerg Med.

[25] Besse M, Wiltfang J, Belz M, Signerski-Krieger J (2022). Einführung digitaler Lehre im Fach Psychiatrie als Reaktion auf COVID-19: eine vergleichende Evaluation zur Präsenzlehre. Nervenarzt.

[26] Cooper MB, Chacko M, Christner J (2018). Incorporating LGBT Health in an Undergraduate Medical Education Curriculum Through the Construct of Social Determinants of Health. MedEdPORTAL.

[27] Grosz AM, Gutierrez D, Lui AA, Chang JJ, Cole-Kelly K, Ng H (2017). A Student-Led Introduction to Lesbian, Gay, Bisexual, and Transgender Health for First-Year Medical Students. Fam Med.

[28] Kelley L, Chou CL, Dibble SL, Robertson PA (2008). A critical intervention in lesbian, gay, bisexual, and transgender health: knowledge and attitude outcomes among second-year medical students. Teach Learn Med.

[29] Barrett DL, Supapannachart KJ, Caleon RL, Ragmanauskaite L, McCleskey P, Yeung H (2021). Interactive Session for Residents and Medical Students on Dermatologic Care for Lesbian, Gay, Bisexual, Transgender, and Queer Patients. MedEdPORTAL.

[30] McKenzie ML, Forstein DA, Abbott JF, Buery-Joyner SD, Craig LB, Dalrymple JL, Graziano SC, Hampton BS, Page-Ramsey SM, Pradhan A, Wolf A, Hopkins L (2020). Fostering Inclusive Approaches to Lesbian, Gay, Bisexual, and Transgender (LGBT) Healthcare on the Obstetrics and Gynecology Clerkship. Med Sci Educ.

[31] Khaw C, Raw L (2016). The outcomes and acceptability of near-peer teaching among medical students in clinical skills. Int J Med Educ.

[32] Bundesärztekammer (2021). Ärztestatistik zum 31. Dezember 2020.

[33] Raupach T, Munscher C, Beissbarth T, Burckhardt G, Pukrop T (2011). Towards outcome-based programme evaluation: using student comparative self-assessments to determine teaching effectiveness. Med Teach.

